# Effects of drought, disturbance, and biotic neighborhood on experimental tree seedling performance

**DOI:** 10.1002/ece3.10413

**Published:** 2023-08-15

**Authors:** Benjamin S. Ramage, Daniel J. Johnson, David M. Chan

**Affiliations:** ^1^ Biology Department Randolph‐Macon College Ashland Virginia USA; ^2^ School of Forest, Fisheries, & Geomatics sciences University of Florida Gainesville Florida USA; ^3^ Department of Mathematics and Applied Mathematics Virginia Commonwealth University Richmond Virginia USA

**Keywords:** conspecific inhibition, conspecific negative density dependence, forest biodiversity, interannual variation, tree species coexistence

## Abstract

Forest biodiversity is likely maintained by a complex suite of interacting drivers that vary in importance across both space and time. Contributing factors include disturbance, interannual variation in abiotic variables, and biotic neighborhood effects. To probe ongoing uncertainties and potential interactions, we investigated tree seedling performance in a temperate mid‐Atlantic forest ecosystem. We planted seedlings of five native tree species in mapped study plots, half of which were subjected to disturbance, and then monitored seedling survival, height growth, and foliar condition. The final year of data collection encompassed a drought, enabling comparison between intervals varying in water availability. Seedling performance was analyzed as a function of canopy cover and biotic neighborhood (conspecific and heterospecific abundance), including interactions, with separate generalized linear mixed models fit for each interval. All species exhibited: (a) pronounced declines in height growth during the drought year, (b) detrimental effects of adult conspecifics, and (c) beneficial effects of canopy openness. However, despite these consistencies, there was considerable variation across species in terms of the relevant predictors for each response variable in each interval. Our results suggest that drought may strengthen or reveal conspecific inhibition in some instances while weakening it or obscuring it in others, and that some forms of conspecific inhibition may manifest only under particular canopy conditions (although given the inconsistency of our findings, we are not convinced that conspecific inhibition is critical for diversity maintenance in our study system). Overall, our work reveals a complex forest ecosystem that appears simultaneously and interactively governed by biotic neighborhood structure (e.g., conspecific and/or heterospecific abundance), local habitat conditions (e.g., canopy cover), and interannual variability (e.g., drought).

## Graphical abstract

Forest biodiversity is likely maintained by a complex suite of linked variables. To probe ongoing uncertainties and potential interactions, we investigated tree seedling performance via a multi‐year, manipulative field experiment. Our work reveals a complex temperate forest ecosystem that appears simultaneously and interactively governed by biotic neighborhood structure (e.g., conspecific and heterospecific abundance), local habitat conditions (e.g., canopy cover), and interannual variability (e.g., drought).

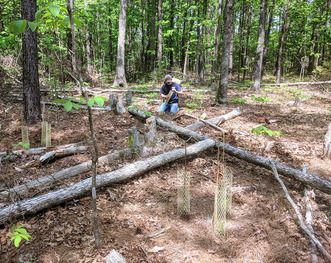

## 1 INTRODUCTION

Forests are among the most complex and speciose ecosystems on Earth. Despite decades of research, questions remain about how forest biodiversity is maintained (Ricklefs, 2015; Keil & Chase, 2019; Sabatini et al., 2022). Why are most forests composed of complex mixtures of closely intermingled tree species (Pommerening & Uria‐Diez, 2017), as opposed to monospecific stands of the tree species best adapted to local conditions? Several explanations have been proposed, and contributing factors likely include biotic neighborhood effects (including conspecific inhibition), disturbance, and interannual variation in abiotic variables like precipitation (Clark et al., 2016; Gravel et al., 2010; Hülsmann et al., 2021).

Conspecific inhibition, a form of species‐specific negative feedback, refers to a reduction in performance (e.g., survival or growth rates) when conspecific densities are high. Inhibition can result from the local accumulation of species‐specific natural enemies (e.g., pathogens, insects) (Comita et al., 2014; Terborgh, 2012), or when intraspecific competition is more intense than interspecific competition (e.g., a tree species with shallow roots will be inhibited more by conspecifics than by a deeply rooted species) (Adler et al., 2018; Rog et al., 2021). Regardless of the precise mechanism, conspecific inhibition prevents or reduces local dominance by one or a few species, thus enhancing local diversity (Hülsmann et al., 2021). In addition, as postulated by the Janzen‐Connell Hypothesis, which asserts that tropical forest diversity is maintained by especially strong conspecific inhibition (Connell, 1971; Janzen, 1970), local impacts may also influence regional diversity patterns (Johnson et al., 2012; Terborgh, 2012). However, conspecific inhibition does not necessarily explain diversity beyond very local scales, and thus it can be an important driver of compositional patterns even in temperate systems with low regional species diversity (Hille Ris Lambers et al., 2002; Hülsmann et al., 2021; Packer & Clay, 2000; Zhu et al., 2015).

Disturbances, discrete events that kill or remove biomass and change abiotic conditions, can be natural or anthropogenic (e.g., hurricane or timber harvest), abiotic or biotic (e.g., wildfire or disease outbreak), and highly variable in a wide range of parameters (Pickett & White, 1986; Sturtevant & Fortin, 2021). Disturbances alter communities directly via mortality as well as indirectly via environmental modification (e.g., increased light and lower humidity) (White & Jentsch, 2001). Biotic disturbances and conspecific inhibition may overlap in certain contexts (e.g., host‐specific pest outbreaks), but disturbances are abrupt and episodic, while conspecific inhibition is usually gradual and ongoing (Canelles et al., 2021; Hülsmann et al., 2021; Nakashizuka, 2001). In addition, disturbance effects are typically much more obvious than those associated with conspecific inhibition. Due to their inherent variability, disturbances almost always increase spatial heterogeneity and beta diversity (White & Jentsch, 2001). Disturbances may also increase local diversity (e.g., by reducing densities of a dominant competitor), but the opposite can occur if the disturbance homogenizes local species composition (e.g., by eliminating fire‐sensitive species in high‐intensity burn patches; Johnson & Miyanishi, 2001).

Abiotic environmental variables exhibit recurring seasonal patterns as well as interannual variability. Predictable seasonal variation is unlikely to serve as a mechanism for maintaining local diversity of long‐lived species, but year to year variation may play an important role via a temporal storage effect (Kelly & Bowler, 2002; Usinowicz et al., 2017). Interannual fluctuations in both means and extreme values may favor different species in different years, increasing the number of species that can competitively coexist (Brodribb et al., 2020; Grubb, 1977; Hansen et al., 2022; Hu et al., 2017). For instance, drought can prove fatal to young seedlings of some tree species, while excess moisture inhibits young seedlings of other species (Clark et al., 2016). Thus, if precipitation is variable across years, more species will eventually experience suitable regeneration windows (Grubb, 1977; Silvertown et al., 2015). The role of interannual climate variability in forest compositional dynamics and diversity maintenance has received less attention than other factors like biotic interactions and microsite conditions (Xu et al., 2022), which is unfortunate given that climate change is likely increasing interannual variation, including drought occurrence and/or magnitude (Brodribb et al., 2020; Clark et al., 2016; Hansen et al., 2022).

Beyond the independent effects of conspecific inhibition, disturbance, and interannual environmental variability, these drivers likely interact with each other. Disturbances can alter a wide range of environmental conditions (e.g., light and soil moisture), and many of these abiotic variables have the potential to affect biological agents of conspecific inhibition (e.g., host‐specific pathogens and insects) as well as plant defenses or other responses (Edmonds et al., 2005; Kardol et al., 2013; Pickett & White, 1986). Pathogenic microbes, which represent a major component of species‐specific mortality agents, generally benefit from the shade and humidity beneath closed canopies (Castello et al., 1995; Mangan et al., 2010). In addition, seedling growth rates often increase following disturbance (more light, less competition), and vigorously growing seedlings are typically more resistant to attack by insects and pathogens. If species‐specific damaging agents are prevalent, these boosted defenses should yield the greatest benefit to seedlings growing near conspecifics (Augspurger & Kelly, 1984; Kardol et al., 2013; McCarthy‐Neumann & Ibáñez, 2013; Reinhart et al., 2010). As such, due to effects on seedlings and/or their enemies, conspecific inhibition may be weakened post‐disturbance (or in patches with greater light availability), and several studies support this trend (Brown et al., 2021; Comita et al., 2009; Esch & Kobe, 2021; Holík et al., 2021; McCarthy‐Neumann & Ibáñez, 2012, 2013). However, conceptual arguments exist for a wide range of light‐induced responses (Smith‐Ramesh & Reynolds, 2017), and there is evidence for the opposing pattern: stronger conspecific inhibition in the wake of disturbance, or in areas with greater light availability (e.g., Magee et al., 2021; Uriarte et al., 2018). This pattern could manifest if, for instance, seedlings experience greater water stress in sunnier areas, and this stress weakens defenses against species‐specific enemies (Desprez‐Loustau et al., 2006). Given these conflicting arguments, it is perhaps not surprising that some studies have found mixed results (e.g., Nagendra & Peterson, 2015).

Interannual environmental variation can also influence conspecific inhibition, and for reasons that broadly align with the causes of disturbance‐related interactions: temperature and moisture affect pests and pathogens as well as plant defenses (Augspurger & Kelly, 1984; Edmonds et al., 2005; Song et al., 2018). The effects of any perturbation—as well as of a general increase in variability—are hard to predict, with neighborhood dynamics likely responding in different ways to different climatic variables (Xi et al., 2022; Xu et al., 2022). Several studies have examined how conspecific effects are influenced by precipitation, one of the most consequential manifestations of interannual variation. Like disturbance, drought has the potential to weaken conspecific inhibition via a reduction of moisture‐loving pathogens or strengthen conspecific inhibition via diminished defenses in water‐stressed plants (Clark et al., 2016; Desprez‐Loustau et al., 2006). In addition, drought can increase negative conspecific effects if intraspecific competition becomes disproportionately impactful in dry conditions (Song et al., 2018). Despite the theoretical possibility for both directions of effect, to the best of our knowledge all relevant publications presenting significant effects have documented weaker conspecific inhibition in drier years (e.g., Bachelot et al., 2015; Newbery & Stoll, 2013; Uriarte et al., 2018; Xi et al., 2022).

Directly documenting diversity maintenance is impractical with long‐lived organisms like trees, so short‐term performance measures are often used to approximate forest compositional trajectories. Observational studies can readily focus on any life stage, ranging from mature trees (e.g., Canham et al., 2006) to saplings (e.g., Ramage et al., 2017) to seedlings (Johnson et al., 2014) and even seeds (Umaña et al., 2016), but experimental research is subject to additional constraints, particularly if the goal is to minimize confounding factors by randomly assigning subjects to microsites. Seedlings are especially useful in this context, as they can be grown in controlled conditions and then transplanted to areas that vary in factors such as light availability and conspecific abundance. In addition, seedlings are among the most fragile and sensitive of tree life stages (Grubb, 1977), and likely the most susceptible to conspecific inhibition (Zhu et al., 2015), which makes it easier to detect effects of interest. While many studies investigating these themes have analyzed seedlings, most have monitored naturally occurring seedlings, making it impossible to fully exclude potentially confounding influences.

Our initial objectives were to investigate how tree seedling performance in a temperate mid‐Atlantic forest ecosystem is interactively affected by two key drivers of forest biodiversity: disturbance and conspecific inhibition. To begin our study, we propagated five native tree species and planted seedlings in mapped study plots, half of which we had previously subjected to disturbance (tree cutting), and then monitored the seedlings for two and a half years. Fortuitously, a drought occurred during the final year, allowing us to incorporate drought effects, and relationships with other predictor variables, into our ultimate objectives. We analyzed seedling performance as a function of canopy cover and biotic neighborhood, and then compared results across pre‐drought and drought‐affected intervals.

## 2 METHODS

All fieldwork was conducted at two field sites in central Virginia, United States. The region is dominated by temperate forest comprised of both hardwoods (e.g., oaks) and conifers (e.g., pines). Summers are hot, winters are moderate, and precipitation is usually plentiful throughout the entire year. At one field site (37.835480, −77.499165), all study plots were in mature (approx. 90 years old), naturally regenerated secondary forest dominated by native deciduous trees, with the overstory composed primarily of oaks (*Quercus* spp.) and yellow poplar (*Liriodendron tulipifera*), and the lower canopy layers including substantial quantities of hickory (*Carya* spp.), sweetgum (*Liquidambar styraciflua*), tupelo (*Nyssa sylvatica*), American holly (*Ilex opaca*), American beech (*Fagus grandifolia*), red maple (*Acer rubrum*), and flowering dogwood (*Cornus florida*). Mean basal area (BA) and stem counts per hectare were 36.5 m^2^ and 1091 trees, respectively, calculated from all trees ≥5 cm diameter at breast height (DBH). The other field site (37.837219, −77.637958), about 8 km from the first, had a sparse overstory of large loblolly pines (*Pinus taeda*) that were planted in the early 1970s and thinned in the early 1990s, with a well‐developed lower canopy of naturally occurring native trees including *Quercus* spp., *L*. *styraciflua*, black cherry (*Prunus serotina*), *N. sylvatica, A. rubrum*, and *I. opaca*. Mean BA and stem counts per hectare were 29.1 m^2^ and 1505 trees, respectively, calculated from all trees ≥5 cm DBH.

Study plots (32 total) were distributed across both study sites in the summer of 2017. Within each plot (17.9 m radius; 1/6 hectare), all trees ≥5 cm DBH were mapped and identified to species. Next, in each plot, we marked and mapped 15 seedling *stations* (sensu Fricke & Wright, 2017) distributed within 8 m of plot center in areas relatively free of understory vegetation and debris (480 stations total across 32 plots). To investigate disturbance effects and interactions, half of the plots at each site were subjected to artificial disturbance treatments (selective cutting of lower canopy trees) in the late summer and fall of 2017. Our intention was not to mimic any particular natural disturbance, but rather to create conditions (e.g., a range of light levels and disturbance legacies) that would facilitate a granular investigation of seedling performance. While we did not cut upper canopy trees, primarily due to safety concerns, removal of the lower canopy layer markedly decreased canopy cover, creating variation in light availability, a critical driver of tree species coexistence (Gravel et al., 2010). Mean canopy openness (1 minus proportion canopy cover) increased from 0.03 in control plots to 0.14 in disturbed plots, and more notably, the 90th percentile of canopy openness increased from 0.05 to 0.26; histograms are provided in the Figure S1 (Supporting Information) to enable a more thorough comparison. On average, from each plot we removed 29 trees, with a mean DBH of 9.1 cm. All tree species were targeted for removal, with the most frequently cut including *Quercus* spp., *Carya* spp., *I. opaca*, *N. sylvatica*, *F. grandifolia*, and *L. styraciflua*. For focal species (those for which seedlings were planted), approximately half of the trees we could have cut were left intact. This approach allowed us to investigate how seedlings are affected by both living and cut conspecifics in their vicinity, and to examine how conspecific effects interact with total canopy cover.

**FIGURE 1:**
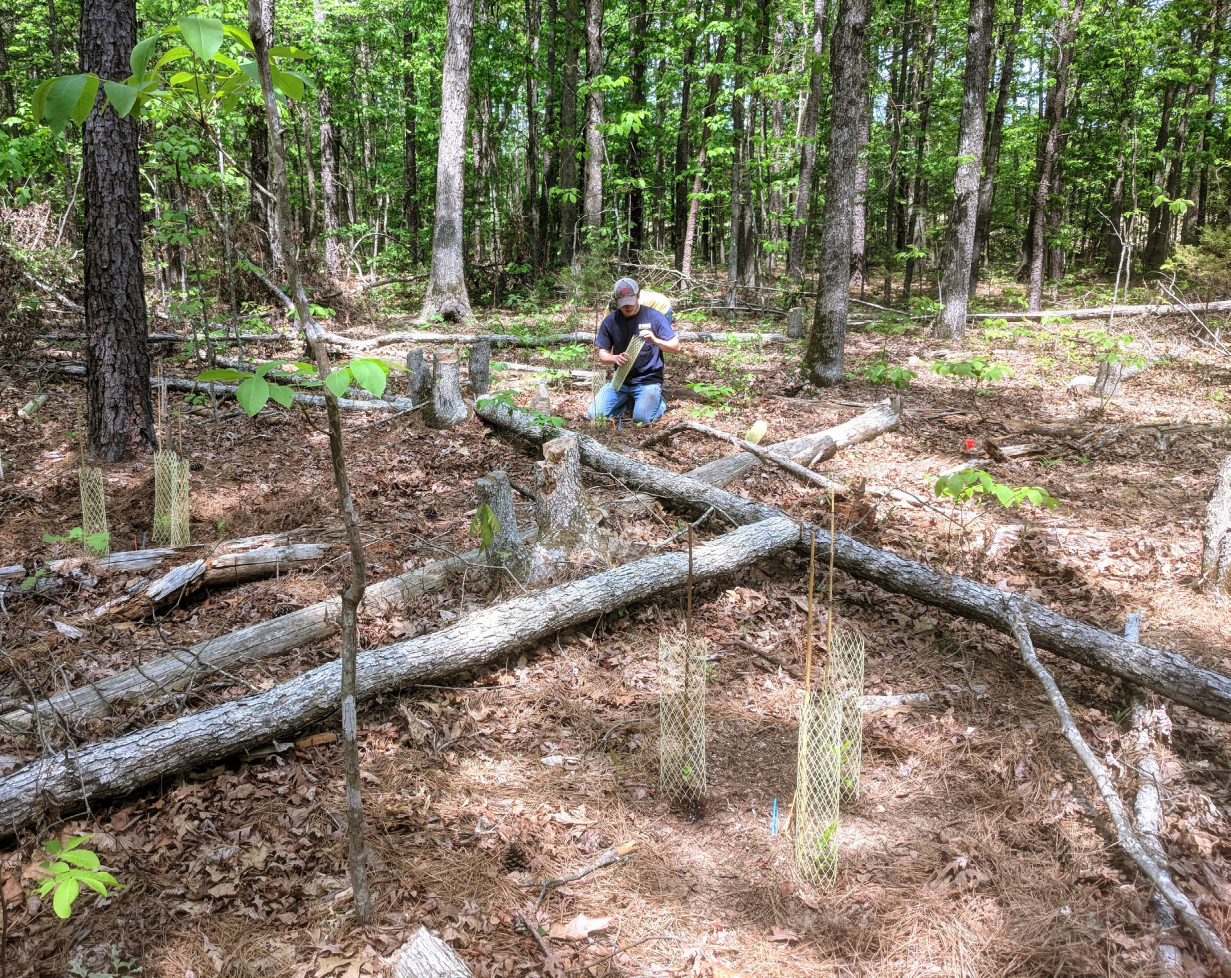
Seedlings planted at seedling stations in a plot that was subjected to disturbance.

Five tree species native to our study region were cultivated and planted at seedling stations: flowering dogwood (*C. florida*), sweetgum (*L. styraciflua*), tupelo (*N. sylvatica*), black cherry (*P. serotina*), and red maple (*A. rubrum*). Seeds were purchased from Sheffield's Seed Company (Locke, NY, United States), and cold‐stratified in the fall of 2017. Seeds were container‐sown indoors in winter 2018 and then planted in study plots in spring 2018. Germination success varied considerably across species, so the total number of seedlings planted in the field ranged from 72 (*A. rubrum*) to 456 (*N. sylvatica*). All seedlings were planted at mapped seedling stations (three per station; 1440 total seedlings), arranged in a triangular pattern, with approximately 30–40 cm separation between seedlings, and each seedling belonging to a different species (Figure 1). Seedlings were protected with mesh herbivore exclusion guards that prevent mammalian herbivory but allow access to insects (which may contribute to conspecific inhibition; Bagchi et al., 2014; Fricke et al., 2014). Water was provided at time of initial planting, but additional water was not needed given the ample rainfall in spring 2018 (Table 1). Canopy cover above each seedling station was measured in summer 2018 with a spherical densiometer.

**TABLE 1:** Project timeline and growing season standardized precipitation and evapotranspiration index (SPEI) values.

	2018			2019			2020		
	April–May	June–July	August–September	April–May	June–July	August–September	April–May	June–July	August–September
SPEI	1.7	1.0	1.2	0.0	−0.1	−1.1	1.1	−0.1	1.8
Activities & data	↑ Field Planting	↑ Survival, Height	↑ Survival, Height, Foliage			↑ Survival, Height, Foliage			↑ Survival, Height
Analysis intervals		—first summer—							
			—pre‐drought—						
						—spanning drought—			

*Note*: Activities that occurred prior to field planting (e.g., disturbance treatments, indoor seed propagation) are not shown here. Arrows indicate timing of data collection or other activities; for instance, 2020 data collection occurred in late July/early August. SPEI data were computed over 2‐month intervals and extracted from https://spei.csic.es/spei_database.

Planted seedlings were initially surveyed in early June 2018 and then monitored periodically until the summer of 2020 (Table 1). In early June 2018, we recorded seedling height and survival, which was 100% at this point. In early August (2 months later), we remeasured height and survival, and recorded foliar damage. Foliar damage symptoms were assessed for the entire seedling (not a subset of leaves) and quantified in several categories (leaf spots, chew holes, etc.), using modified Braun‐Blanquet cover classes. All damage categories were summed for the analysis presented here. In summer 2019 (late July/early August), hereafter just “2019”, we remeasured survival, height, and foliar damage. In Summer 2020 (late July/early August), hereafter just “2020”, we once again recorded survival and height, but we were unable to re‐assess foliar damage during this final data collection effort due to complications related to the COVID disruption.

Seedling performance was analyzed via generalized linear mixed models, with separate models fit for each species, analysis interval (Table 1), and measure of performance (survival, height growth, and foliar damage). We have not included community‐level models because the wide range in sample sizes across species would lead to the more abundant species dominating the results. Survival models, with a binomial error distribution (and logit link function), were fit for the Aug 2018–2019 & 2019–2020 intervals; survival from Jun 2018 to Aug 2018 was not analyzed because nearly 100% of seedlings survived. Height growth models, with a Gaussian error distribution, were fit for all intervals: Jun 2018–Aug 2018, Aug 2018–2019, and 2019–2020. Foliar damage models, with a Poisson error distribution (and log link function), were fit for Aug 2018 and 2019 measurements; as noted above, foliar damage was not quantified in 2020.

Fixed effect predictors in all models were: (a) initial height (measured in June 2018), which is often included as a covariate to account for size‐dependent survival or growth (e.g., Johnson et al., 2017; Xu et al., 2022), (b) canopy openness, and (c) local abundance (inverse distance weighted basal area, IDW BA, within a 10 m radius of each station) of living conspecifics, cut conspecifics (to look for potential lag effects; Esch & Kobe, 2021), and living heterospecifics. In addition, we included interactions between canopy openness and conspecific abundance (living and cut, separately), matching the approach used by Holík et al. (2021), and similar to that of Xu et al. (2022), who modeled interactions between conspecific abundance and annually fluctuating climatic variables.

Predictors were standardized, separately for each model subset, by subtracting the mean and dividing by the standard deviation. All variance inflation factors were below five for all significant predictors in all models; while some interaction terms yielded higher values in some models (between five and ten), none of the affected variables were found to have significant effects. In all models, field site and plot were specified as nested random effects. All models were fit with the function “glmmPQL” in the R package *MASS* (Venables & Ripley, 2002), and IDW BA calculations were done with assistance from R package *spatstat* (Baddeley et al., 2015).

To examine drought effects, we compare models for pre‐drought and post‐drought intervals. Rainfall was abundant in 2018 and average in the early summer of 2019 but pronounced drought conditions developed in the late summer and early fall of 2019 (Table 1), after 2019 data collection. As such, the 2018–2019 analysis interval was characterized by sufficient rainfall throughout the growing season, while the 2019–2020 interval encompasses the drought conditions that manifested in August and September of 2019. Across these 2 months, the SPEI (Standardized Precipitation and Evapotranspiration Index; https://spei.csic.es) was −1.1 (Table 1). Both intervals span 1 year (approximately), so large differences in survival or height growth are likely due to differences in water availability. Foliar damage was not measured post‐drought, so we are unable to look at pre‐ versus post‐drought comparisons for this response variable.

## 3 RESULTS

Survival rates for all species were high (≥0.94) through the first summer (June 2018–August 2018) and moderate (*≥*0.82) through the first full‐year interval (August 2018–2019), which was not affected by the drought (Table 2). In the second full‐year interval (2019–2020), which encompassed drought conditions, survival rates dropped considerably for some species. Drought‐associated survival declines were most apparent for *C. florida* (0.82 ➔ 0.65) and *N. sylvatica* (0.84 ➔ 0.63). Smaller declines were observed for *P. serotina* (0.86 ➔ 0.78) and *A. rubrum* (0.93 ➔ 0.87), and survival was essentially unchanged for *L. styraciflua* (0.84 ➔ 0.83).

**TABLE 2:** Survival rates and height growth means for all analysis intervals.

	# planted	First summer (6/18–8/18)		Pre‐drought (2018–2019)		Spanning drought (2019–2020)	
		Survival	Height growth (cm)	Survival	Height growth (cm)	Survival	Height growth (cm)
*Cornus florida*	223	0.94		0.82		0.65	
			1.80		5.96		2.86
*Liquidambar styraciflua*	390	0.98		0.84		0.83	
			0.16		6.65		4.23
*Nyssa sylvatica*	456	0.96		0.84		0.63	
			2.01		6.59		0.45
*Prunus serotina*	236	1.0		0.86		0.78	
			−0.14		6.53		1.52
*Acer rubrum*	72	1.0		0.93		0.87	
			2.07		6.67		2.42

Note that the “first summer” values are not directly comparable to the other two intervals because it spans only 2 months; in contrast, the “pre‐drought” and “spanning drought” intervals both span an entire year. Survival rates are not cumulative but rather the proportion that survived from the start of the interval.

Mean growth rates were universally lower, and in some cases dramatically so, for the interval that encompassed the drought (Table 2). *N. sylvatica* height growth nearly ceased, falling from 6.59 cm in the pre‐drought interval (2018–2019) to 0.45 cm during the drought year (2019–2020), a 93% reduction. Notable declines across the same two intervals were also observed for *P. serotina* (6.53 cm ➔ to 1.52 cm, a 77% drop), *A. rubrum* (6.67 cm ➔ 2.42 cm, 64% down), and *C. florida* (5.96 cm ➔ 2.86 cm, a 52% decline). Even *L. styraciflua*, which appears to be the least affected species overall, experienced a 36% reduction in mean growth rates (6.65 cm ➔ 4.23 cm). First summer height grown means are provided for reference (Table 2), but since this interval is much shorter than the other two, comparisons would not be meaningful.

We now turn to the results of our generalized linear mixed models, with each response variable considered in turn and examined across species and analysis intervals. Given the very high survival rates through the first summer (≥0.94 for all species), survival models were fit only for the two full‐year intervals, while height growth models were fit for all three intervals. Foliar damage was not measured in 2020, so models for this response variable are provided only for August 2018 and 2019. An alternative presentation, with each species discussed individually and comprehensively, is provided in the Supporting Information.

**FIGURE 2:**
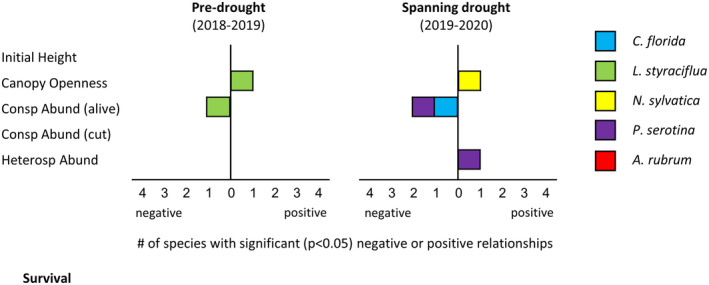
Drivers of seedling survival, summarized across species. As an example, in the year that encompassed the drought (2019–2020), survival was negatively associated with living conspecific abundance for both *Cornus florida* and *Prunus serotina*. Complete survival model results for each species, including interaction terms, are provided in Figure S2 (Supporting Information).

Seedling survival was most consistently affected by living conspecific adults, with three species (*C. florida*, *L. styraciflua*, and *P. serotina*) exhibiting a negative response (Figure 2 and Figure S2). The detrimental conspecific effect varied across analysis intervals, with *C. florida* and *P. serotina* affected only during the drought‐affected year, and *L. styraciflua* affected only during the pre‐drought year. No positive effects of living conspecific abundance on seedling survival were detected and cut conspecific abundance was non‐significant. Heterospecific abundance was mostly non‐significant, the sole exception being a positive effect on *P. serotina* survival during the drought year. Canopy openness increased seedling survival for *L. styraciflua* during the pre‐drought year and for *N. sylvatica* during the drought year, but neither species was affected during the other interval, and no other species exhibited relations between canopy openness and survival.

**FIGURE 3:**
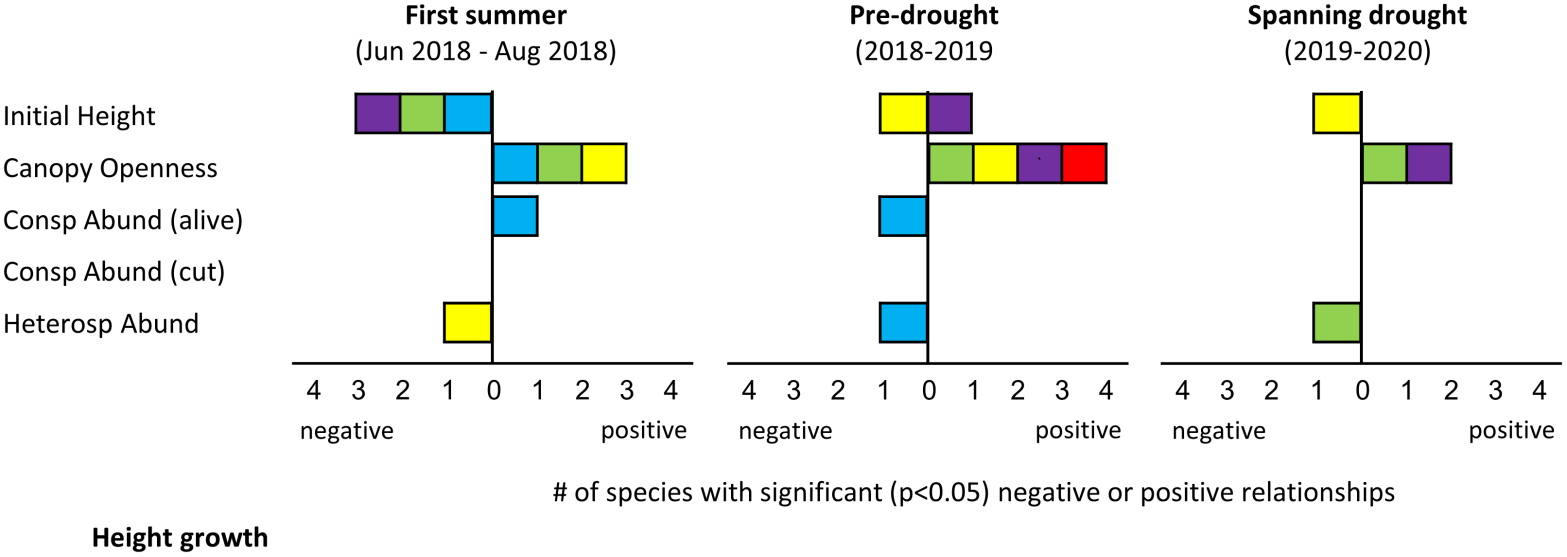
Drivers of seedling height growth, summarized across species. Species color‐coding follows Figures 2 and 4. Complete height growth model results for each species, including interaction terms, are provided in Figure S3 (Supporting Information).

Height growth was strongly linked to canopy openness (Figure 3 and Figure S3). All five species exhibited positive height growth responses to canopy openness in at least one interval, with no negative impacts, although effects were variable across intervals. Canopy openness increased height growth for *C. florida* during the first summer only, *L. styraciflua* during all three intervals, *N. sylvatica* during the first summer and pre‐drought year, *P. serotina* during both full‐year intervals, and *A. rubrum* during the pre‐drought year only. Initial height also proved to be an important predictor of subsequent height growth, with four species exhibiting evidence of reduced growth for initially taller seedlings. These effects were limited to the first summer for *C. florida*, *L. styraciflua*, and *P. serotina*, and to the two full‐year intervals for *N. sylvatica*. For *P. serotina*, however, the negative effect during the first summer switched to a positive effect (initially taller seedlings growing faster) during the pre‐drought year. Seedling height growth was mostly unaffected by living conspecific abundance, with only *C. florida* exhibiting an apparent relationship, and the effects reversing across intervals (positive during the first summer and negative during the pre‐drought year). Three species were negatively affected by heterospecific abundance, but for each the effect was limited to one interval (the pre‐drought year for *C. florida*, the drought year for *L. styraciflua*, and the first summer for *N. sylvatica*).

Cut conspecific abundance was never consequential as a solo predictor of seedling height growth, but interactions with canopy openness were significant in some species‐specific models (Figure S3). For *L. styraciflua* during the pre‐drought year, cut conspecific abundance was positively linked to height growth in open canopy conditions, but negatively linked to height growth in less open conditions. For *N. sylvatica*, canopy openness interacted with cut conspecific abundance in the first summer and pre‐drought year, but the direction of the interaction differed across these two intervals. During the summer of 2018, increasing cut conspecific abundance led to *decreased* growth rates in areas with less canopy openness, but from 2018 to 2019, increasing cut conspecific abundance led to *increased* growth rates under more closed canopies. In both cases, cut conspecific abundance was predicted to have little or no effect in more open areas. However, because the range of cut conspecific abundance in shadier areas is small, we are limited in our ability to interpret these results.

**FIGURE 4:**
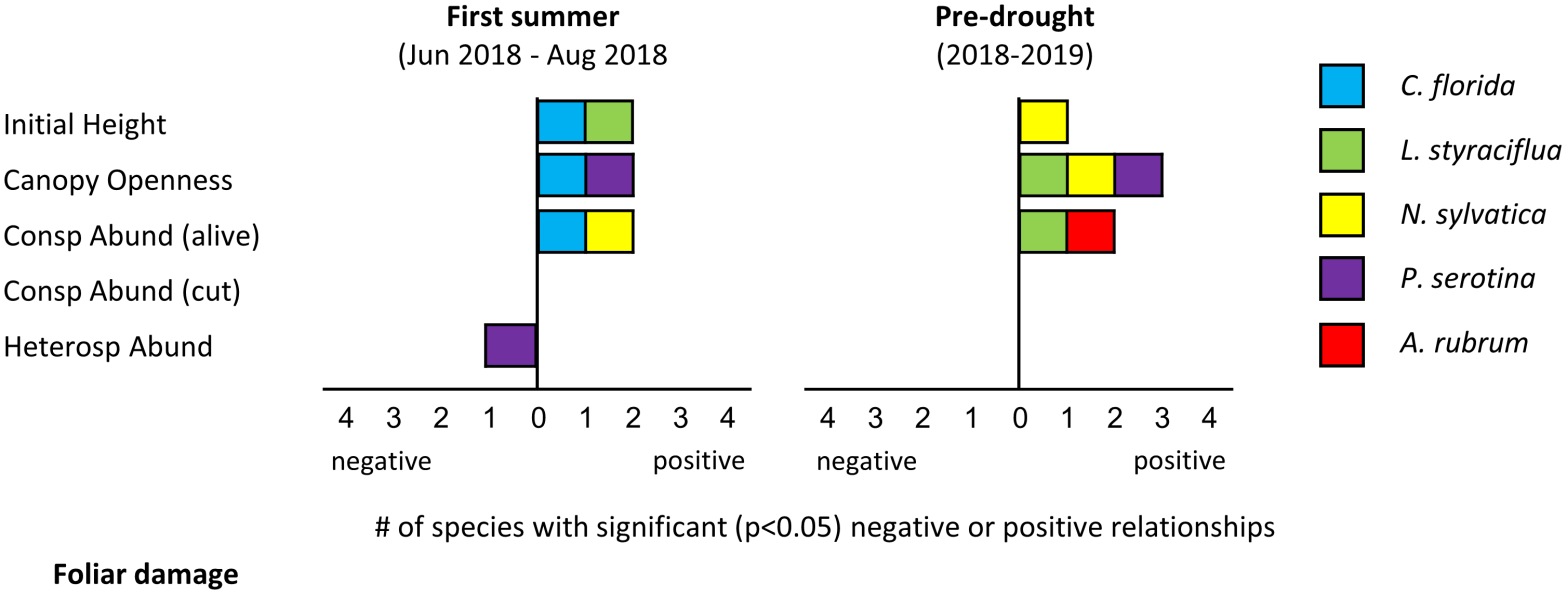
Drivers of seedling foliar damage, summarized across species. Complete foliar damage model results for each species, including interaction terms, are provided in Figure S4 (Supporting Information).

Foliar damage was most related to canopy openness, living conspecific abundance, and initial height (Figure 4 and Figure S4). In all cases for which it was significant, increasing canopy openness was associated with greater foliar damage: *C. florida* during the first summer, *L. styraciflua* and *N. sylvatica* during the pre‐drought year, and *P. serotina* during both intervals. All detectable effects of living conspecific abundance were also detrimental, and again variable across intervals. As the abundance of conspecific neighbors increased, foliar damage increased for *C. florida* and *N. sylvatica* during the first summer, and for *L. styraciflua* and *A. rubrum* during the pre‐drought year. A detrimental effect of initial height was also detected for three species (*C. florida*, *L. styraciflua*, and *N. sylvatica*), with taller seedlings suffering more foliar damage. Heterospecific trees were found to be protective in one instance, with *P. serotina* in the first summer displaying less foliar damage as heterospecific abundance increased. Finally, *N. sylvatica* exhibited a significant interaction between canopy openness and living conspecific abundance in the pre‐drought year; foliar damage was predicted to increase with living conspecific abundance, but only in open areas.

## 4 DISCUSSION

Our results reveal a wide array of responses that vary across species and measurement intervals. At the same time, however, several broader trends are apparent. Perhaps most obvious are the effects of the drought that influenced our final measurement interval (2019–2020). Mean height growth was lower for all species during the drought year, with growth reductions relative to the previous year ranging from 36% (*L. styraciflua*) to an astounding 93% (*N. sylvatica*). Survival rates also declined for all species, although the drop was less pronounced compared to height growth, and some species (e.g., *L. styraciflua*) experienced minimal reductions. Overall, *L. styraciflua* seemed to be the species least affected while *N. sylvatica* proved most drought‐sensitive. Previous research has also suggested that temperate forest tree seedlings may be more sensitive to drought than variation in local habitat or biotic neighborhood conditions (Xu et al., 2022).

We also observed some consistencies related to conspecific effects. Detrimental effects of living conspecific adults were detected for all species, during at least one interval and for at least one response variable, although the potential for false positives is a concern given the total number of tests conducted. In contrast, however, beneficial conspecific effects were present for only one species (*C. florida*), in one interval (the first summer) and one response variable (height growth). While focal species varied in their local abundance averages and ranges (Table S1), this variation does not appear correlated with the likelihood of detecting conspecific effects; all species exhibited at least one significant conspecific effect, and the species for which conspecific effects were most common (*C. florida*) was intermediate in its abundance average and range. However, abundances of all focal species were generally low relative to the basal area of overstory trees (mostly *Quercus* spp., *L. tulipifera*, and *P. taeda*), and thus it is possible that more relationships—negative and/or positive—would have been detectable if our dataset had encompassed patches dominated by conspecifics. These limitations aside, our findings suggest that negative conspecific effects might be more prevalent than positive conspecific effects in the community we investigated. However, we also documented detrimental effects of adult heterospecifics for three of five species, with beneficial heterospecific effects found for only one (*P. serotina*). As such, the apparent negative conspecific effects we documented may represent general competitive inhibition. In addition, short‐term conspecific effects do not necessarily affect longer‐term biodiversity dynamics (Hülsmann et al., 2021), warranting continued long‐term study.

Canopy openness was the most influential predictor overall, but effects were divergent across response variables, with results hinting at a linked conspecific component. Not surprisingly, greater light availability led to increased height growth for all species, although effects were not consistent across measurement intervals. Survival was positively affected by light availability for only two species (*L. styraciflua* and *N. sylvatica*), and not across both intervals. No negative relationships between canopy openness and either height growth or survival were detected. However, despite these universally positive/neutral trends for height growth and survival, four out of five species (*C. florida*, *L. styraciflua*, *N. sylvatica*, and *P. serotina*) had more foliar damage in areas with greater canopy openness, in at least 1 year. Foliar damage was also positively associated with living conspecific abundance for four species (*C. florida*, *L. styraciflua*, *N. sylvatica,* and *A. rubrum*), perhaps indicating the presence of species‐specific foliar pests or pathogens that require adequate light or that more readily find or disperse to plants in less sheltered locations (Martini et al., 2021). Lending support to this speculative interpretation is a significant interaction between canopy openness and living conspecific abundance for *N. sylvatica*; foliar damage was predicted to increase with living conspecific abundance, but only in areas with reduced canopy cover. This finding is similar to that of Magee et al. (2021), which found that seedling survival was influenced by an interaction between canopy openness and conspecific tree density; at high conspecific density, survival was higher in lower light conditions, suggesting that high light and high conspecific abundance may be a dangerous combination in some situations.

Heterospecific trees may have a protective effect in some instances (Peters, 2003; Xu et al., 2022). Like several species, *P. serotina* seedlings were negatively affected by conspecific adults (reduced survival during the drought year), but *P. serotina* was unique in that it also appeared to benefit from proximate heterospecifics, matching the community‐wide results of Xu et al. (2022). Furthermore, this heterospecific signal appeared in two response variables across two intervals. In the first summer (2018), foliar damage was lower where heterospecific abundance was greater, and in the drought year (2019–2020), survival was positively associated with heterospecific abundance. If airborne species‐specific pathogens are important for *P. serotina* seedling survival, then it makes sense that proximate heterospecific foliage would intercept propagules and minimize pathogen exposure, as per the species herd protection hypothesis (Peters, 2003). So, even though *P. serotina* seedlings grew faster in more exposed locations, this exposure might also make them more prone to potentially lethal foliar infections, especially if mature *P. serotina* trees are nearby.

Beyond the direct effects of drought on survival and growth, drought may interact with other drivers of seedling performance (Uriarte et al., 2018; Xi et al., 2022). For three of our seedling species, the effects of canopy openness differed between the drought year and non‐drought intervals. For both *A. rubrum* and *N. sylvatica*, canopy openness was positively associated with height growth prior to the drought, but this predictor became non‐significant over the interval that encompassed the drought, perhaps reflecting a light‐moisture tradeoff that roughly balanced out in dry conditions (Clark et al., 2016; Desprez‐Loustau et al., 2006). A similar pattern was documented for *L. styraciflua*, but for survival instead of height growth. However, the *N. sylvatica* survival trend is puzzling, as it is the opposite of the aforementioned results, including its own height growth trend. Nothing was predictive of *N. sylvatica* survival prior to the drought, but canopy openness was positively associated with survival through the drought period. We cannot confidently explain this pattern, but one possibility is that seedlings with ample exposure were able to grow more expansive root systems prior to the drought, and these well‐developed root systems then facilitated survival during the drought (Harrison & LaForgia, 2019). It is also possible, however, that these fluctuations are effectively random and unrelated to the drought.

Drought may also alter conspecific and heterospecific effects (Clark et al., 2016; Xi et al., 2022), but apparently not in a consistent or predictable manner. Our *L. styraciflua* results, for instance, suggest that drought suppressed conspecific inhibition; *L. styraciflua* survival was negatively affected by living conspecifics pre‐drought, but this effect disappeared during the drought year. Unlike all other species, *L. styraciflua* survival did *not* decline during the drought, making it unlikely that drought‐driven mortality simply obscured the signal of a conspecific effect. Instead, this shift may implicate a species‐specific, moisture‐loving pest or pathogen as a key agent of conspecific inhibition (and likely one that attacks leaves given the aforementioned relationship between conspecific abundance and foliar damage). *L. styraciflua* seedlings were affected by heterospecific trees in a very different way; heterospecific abundance had no significance prior to the drought but became negatively associated with seedling height growth during the drought year, suggesting that perhaps drought conditions were necessary to make interspecific belowground competition relevant (Gleason et al., 2017). In contrast to *L. styraciflua*, our *P. serotina* results indicate that drought may have instigated and/or revealed negative conspecific effects; pre‐drought, *P. serotina* seedling mortality was low and predictor variables had no predictive value, but during the drought year, mortality increased somewhat and living conspecific abundance had a negative effect on survival. Again, however, these suggestions are speculative, and additional research would be needed to draw more definitive conclusions.

Despite clear evidence that seedling performance through the drought‐affected year differed markedly from the previous year, in terms of mean values as well as key drivers, we cannot be certain that all apparent differences were influenced by the drought. While major declines in survival and growth are most parsimoniously explained by a reduction in water availability (Clark et al., 2016; Hu et al., 2017), some of our findings (e.g., shifts in conspecific effects) could be statistical illusions, driven by other fluctuating factors, or even related to seedling age or size. As an example, Holík et al. (2021) found that—in the same year—the survival of smaller seedlings exhibited a weak interaction between conspecific abundance and canopy cover while no such interaction was found for larger seedlings. However, if the patterns we have documented are to be trusted, it is possible to weave together an interesting story, focused on *C. florida*, that links numerous response and predictor variables and spans all analysis intervals (see Supporting Information).

## 5 CONCLUSIONS

Debates persist about the maintenance of biodiversity in temperate forests, especially regarding the importance of conspecific inhibition (Hülsmann et al., 2021). While we documented detrimental conspecific effects for all species, conspecific effects were not especially pronounced or consistent, and only slightly more common than detrimental heterospecific effects. As such, despite that fact that negative conspecific effects must overcome any positive local site effects (e.g., microsite habitat partitioning) to be detectable (Wu et al., 2016), and that some species may be particularly vulnerable (e.g., *C. florida*), we are not convinced that conspecific inhibition is central to diversity maintenance in the seedling filtering stage of the forest ecosystem we studied. Other factors such as light and water availability may have an equal or greater influence on growth and survival (Xu et al., 2022). Since these abiotic factors vary across both space and time, and their effects are not consistent across species, fluctuating compositional patterns should be expected. In summary, our results tell a complex, multi‐causal story of tree seedling performance. Key drivers vary across species, years, and performance metrics, and compositional dynamics appear to be simultaneously and interactively governed by local habitat conditions (e.g., canopy cover), biotic neighborhood structure (e.g., conspecific abundance), and interannual variability (e.g., drought).

## AUTHOR CONTRIBUTIONS

**Benjamin S. Ramage:** Conceptualization (lead); formal analysis (lead); funding acquisition (equal); investigation (lead); methodology (lead); project administration (lead); supervision (lead); writing – original draft (lead); writing – review and editing (equal). **Daniel J. Johnson:** Conceptualization (supporting); formal analysis (supporting); methodology (supporting); writing – review and editing (equal). **David M. Chan:** Conceptualization (supporting); funding acquisition (equal); writing – review and editing (equal).

## CONFLICT OF INTEREST STATEMENT

The authors declare that they have no conflicts of interest.

## Supporting information


Data S1.

## Data Availability

Data and associated R scripts are available via Dryad: doi: 10.5061/dryad.bg79cnpgjpt.
