# Supplementary material for: Effects of drought, disturbance, and biotic neighborhood on experimental tree seedling performance
Source: Ecol Evol. 2023 Aug 15;13(8):e10413. doi: 10.1002/ece3.10413 (PMC10427772; doi:10.1002/ece3.10413)
Supplement: Supplementary file 1 — Data S1. [file ECE3-13-e10413-s001.pdf]

Supporting Information for:

Effects of drought, disturbance, and biotic neighborhood on experimental tree seedling performance

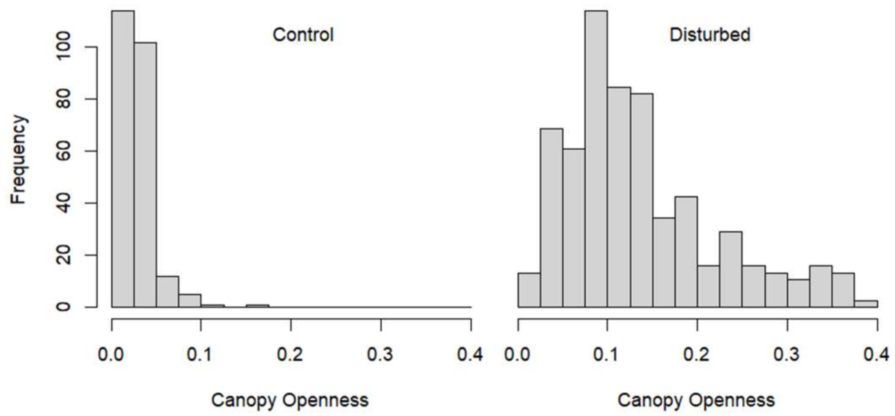

Fig S1. Canopy openness in control and disturbed plots. Observations were taken at each seedling station (15 per plot) with a spherical densiometer. A value of 0 indicates a fully closed canopy and a value of 1 would indicate unbroken sky.

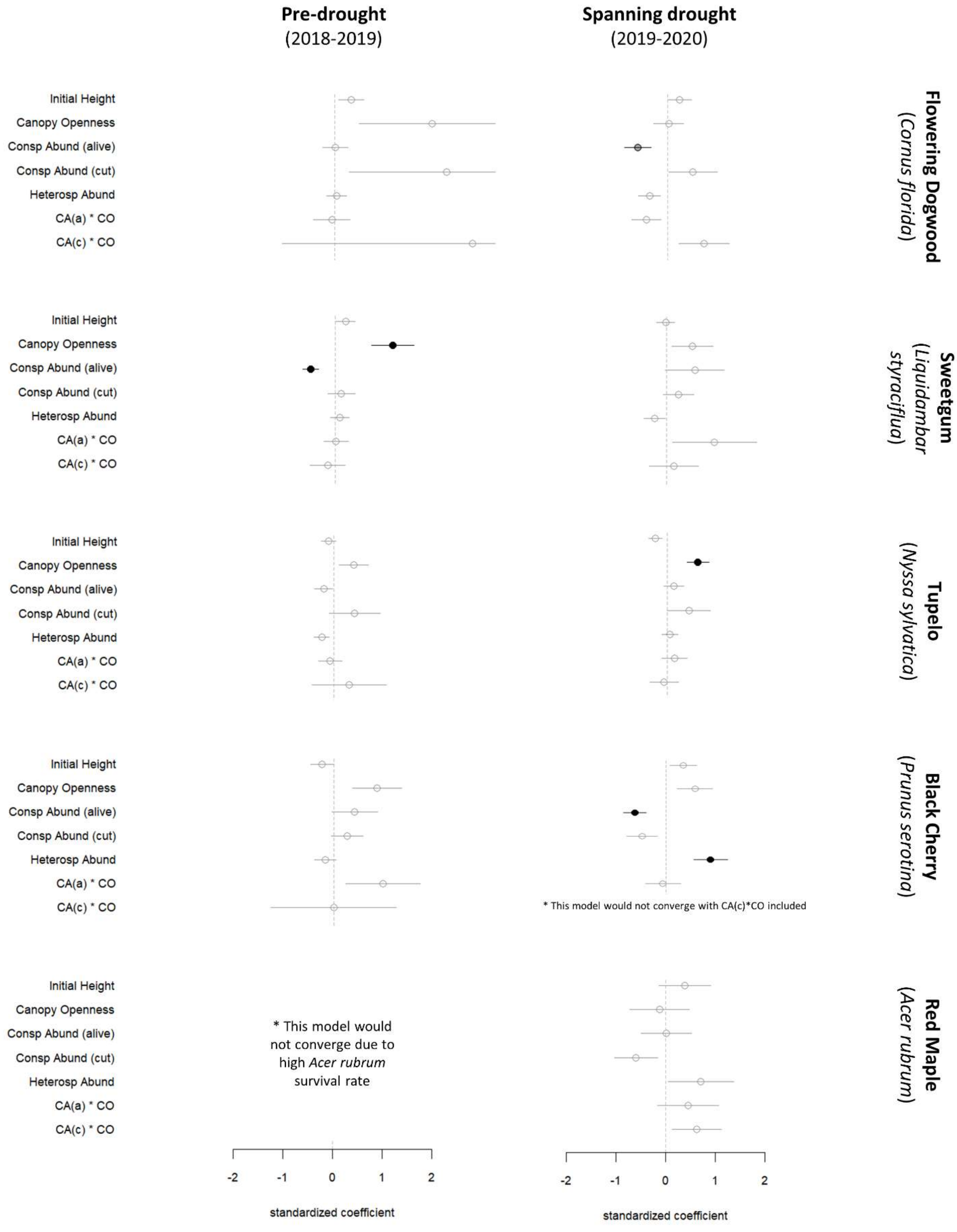

Fig. S2. Drivers of seedling survival in species-specific models. Parameter estimates and SEs are displayed. *CA(a), CA(c)* = conspecific abundance (alive or cut); *CO* = canopy openness. Significance is indicated by symbology: grey border/line = p>0.05; black border/line = p<0.05; black fill = p<0.01.

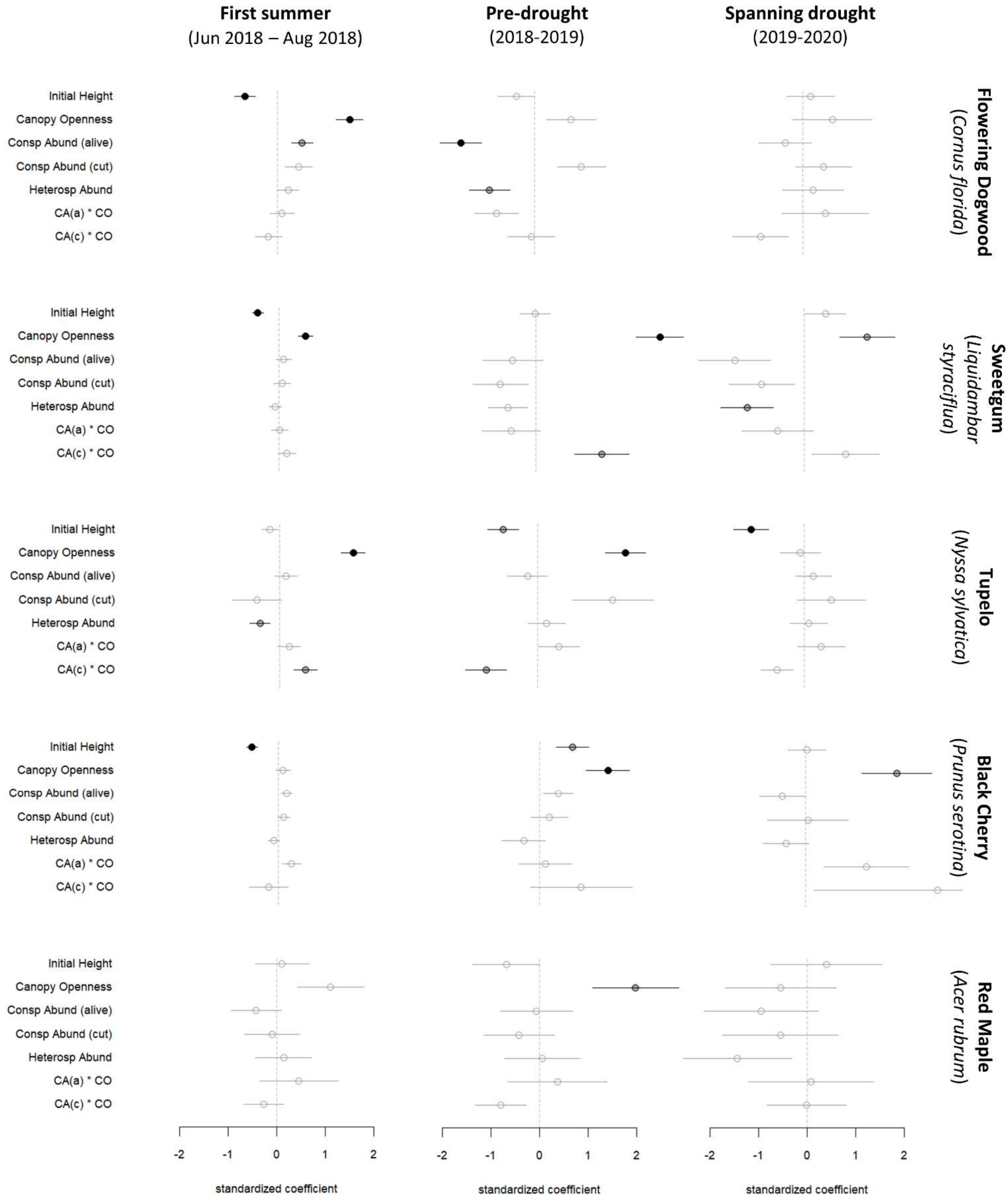

Fig. S3. Drivers of height growth in species-specific models. Parameter estimates and SEs are displayed. *CA(a), CA(c)* = conspecific abundance (alive or cut); *CO* = canopy openness. Significance is indicated by symbology: grey border/line = p>0.05; black border/line = p<0.05; black fill = p<0.01.

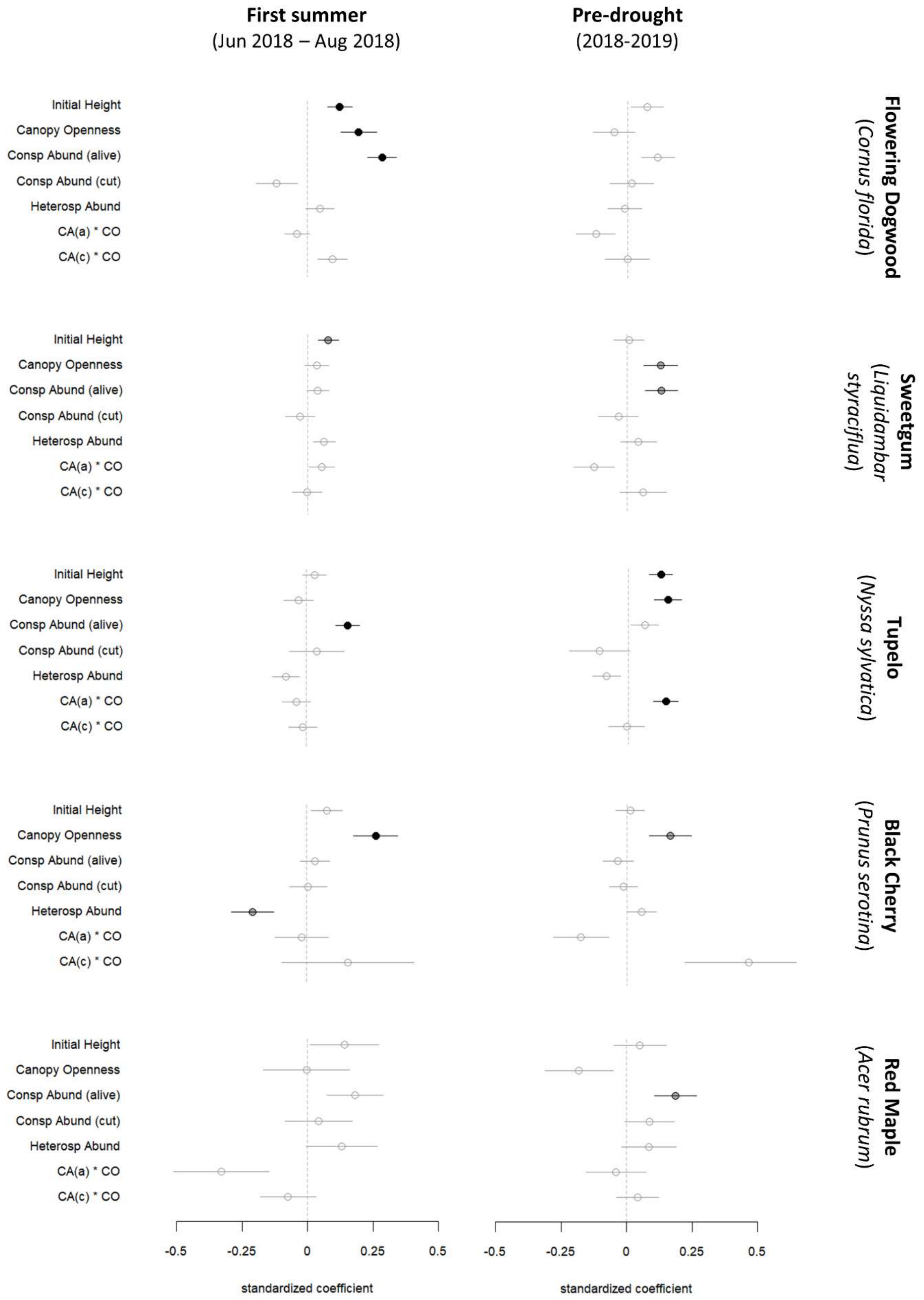

Fig. S4. Drivers of foliar damage in species-specific models. Parameter estimates and SEs are displayed. *CA(a), CA(c)* = conspecific abundance (alive or cut); *CO* = canopy openness. Significance is indicated by symbology: grey border/line = $p>0.05$; black border/line = $p<0.05$; black fill = $p<0.01$.

Table S1. Conspecific abundance within local neighborhoods (10-meter radius) of planted seedlings. Values are medians, with 10th and 90th quantiles provided in parentheses. Because local neighborhoods necessarily overlap, values were first averaged within each plot, and then medians and quantiles were calculated at the plot level. Neighborhood abundance calculations were only done for seedling stations at which each focal species was planted, as these are the neighborhoods subject to conspecific effects analysis. For instance, because *Cornus florida* was not planted at the "Planted pine overstory" site (due to generally low abundance of this species at that site), there were no conspecific neighborhoods to evaluate, hence the "NA" table entries. Total basal area (BA), for both conspecifics and all species, includes living and cut trees.

	**Natural forest**		**Planted pine overstory**	
	Total conspecific BA (m^2)	Consp. proportion of all-species BA	Total conspecific BA (m^2)	Consp. proportion of all-species BA
Cornus florida	0.014 (0.003, 0.033)	0.013 (0.003, 0.041)	NA	NA
Liquidambar styraciflua	0.004 (0.000, 0.344)	0.004 (0.000, 0.352)	0.011 (0.000, 0.056)	0.017 (0.000, 0.076)
Nyssa sylvatica	0.039 (0.013, 0.134)	0.036 (0.010, 0.139)	0.002 (0.000, 0.010)	0.004 (0.000, 0.013)
Prunus serotina	0.000 (0.000, 0.003)	0.000 (0.000, 0.002)	0.011 (0.004, 0.019)	0.013 (0.006, 0.027)
Acer rubrum	0.035 (0.010, 0.067)	0.032 (0.012, 0.079)	NA	NA
**All species**	Total BA (m^2) = 1.056 (0.722, 1.306)		Total BA (m^2) = 0.695 (0.578, 0.940)	

<u>Generalized linear mixed models results, organized by species</u>

Given the very high survival rates through the first summer ($\geq$ 0.94 for all species), survival models were fit only for the two full-year intervals. In addition, foliar damage was not measured in 2020, so models for this response variable are provided only for August 2018 and 2019. Height growth models were fit for all three intervals.

Flowering dogwood (*Cornus florida*) survival was negatively associated with living conspecific abundance in the drought year, but not in the pre-drought year, and was unrelated to all other predictor variables (Fig. S2). *C. florida* height growth was linked to several predictors, but with no consistency across measurement intervals (Fig. S3). In the first summer, height growth was strongly positively associated with canopy openness and negatively associated with initial height. In addition, height growth was slighter greater in areas with higher living conspecific abundance. However, in the pre-drought year, this conspecific effect reversed, becoming strongly negative. In this same interval, heterospecific abundance also had a negative effect on height growth. No model predictors were significantly associated with height growth during the interval that spanned the drought. *C. florida* foliar damage in the first summer was greater for seedlings that were initially taller, in more exposed locations (higher canopy openness), and in areas with greater living conspecific abundance (Fig. S4). In 2019, foliar damage had no apparent relationship with any model predictors.

Sweetgum (*Liquidambar styraciflua*) survival in the pre-drought year was positively associated with canopy openness and negatively associated with living conspecific abundance (Fig. S2). In the year encompassing the drought, no model predictors were linked to *L. styraciflua* survival. *L. styraciflua* height growth was higher in areas with greater canopy openness, and this pattern held across all three measurement intervals (Fig. S3). Height growth was negatively linked to initial height in the first summer, but this predictor had no effect in subsequent intervals. In the drought year, heterospecific abundance had a negative influence on height growth, but no other intervals revealed a heterospecific effect. In the pre-drought year, there was a significant interaction between canopy openness and cut conspecific abundance. Cut conspecific abundance was positively linked to height growth in open canopy conditions, but negatively linked to height growth in less open conditions. *L. styraciflua* foliar damage was greater in the first summer on seedlings that were initially taller, and greater in 2019 on seedlings in locations with more canopy openness and higher living conspecific abundance (Fig. S4).

Tupelo (*Nyssa sylvatica*) survival was higher in areas with greater canopy openness in the drought-affected year, but not in the pre-drought year, and was unrelated to any other predictor variables (Fig. S2). *N. sylvatica* height growth was linked to several model predictors, two of which exhibited significant interactions (Fig. S3). Canopy openness was strongly associated with height growth in the first summer and pre-drought year, but not in the drought year. Initial height was negatively linked to height growth in the pre-drought and drought intervals, but not in the first summer. Locations with greater heterospecific abundance had lower height growth in the first summer, but this predictor had no apparent effect in later intervals. Canopy openness interacted with *cut* conspecific abundance in the first summer and pre-drought year, but the direction of the interaction differed across these two intervals. During the summer of 2018, increasing cut conspecific abundance led to *decreased* growth rates in darker areas, but from 2018 to 2019, increasing cut conspecific abundance led to *increased* growth rates in darker areas. In both cases, cut conspecific abundance was predicted to have little or no effect in more open areas. However, because the range of cut conspecific abundance in darker areas is

limited, we are not comfortable reading much into these results. *N. sylvatica* foliar damage was also associated with several model predictors and an interaction (Fig. S4). In the first summer, higher living conspecific abundance was associated with greater foliar damage. In the pre-drought year, more foliar damage was observed on seedlings that were initially taller and planted in areas with greater canopy openness. Living conspecific abundance had no effect as a solo predictor in the pre-drought year, but there was a significant interaction with canopy openness: foliar damage was predicted to increase with living conspecific abundance, but only in open areas.

Black cherry (*Prunus serotina*) survival was unaffected by all model predictors in the pre-drought year, but – in the drought year – *P. serotina* survival was lower in areas with greater living conspecific abundance and higher in areas with greater heterospecific abundance (Fig. S2). *P. serotina* height growth was positively linked to canopy openness in the pre-drought and drought year (Fig. S3). Initial height was also predictive of height growth, but in an inconsistent manner; initially taller seedlings grew slower the first summer, but faster over the course of the following year. No other predictors were related to height growth. *P. serotina* foliar damage was greater in areas with more canopy openness, both in the first summer and the pre-drought year (Fig. S4). Foliar damage was lower in areas with higher heterospecific abundance, but only in the first summer.

Red maple (*Acer rubrum*) survival was apparently unaffected by the variables we included in our model (Fig. S2). No predictors were significant during the drought year, and the pre-drought survival model would not converge, mostly likely because of the high survival rate through that interval (0.93) and/or the low sample size relative to other species (only 72 seedlings planted). In the pre-drought year, *A. rubrum* height growth was greater in areas with more canopy openness, but this pattern did not manifest for the other intervals, and no other model predictors were significant (Fig. S3). *A. rubrum* foliar damage was greater in areas with higher living conspecific abundance, but in 2019 only (Fig. S4). Foliar damage was unrelated to all other variables. As with survival, the low *A. rubrum* sample size may have obscured some height growth and foliar damage relationships.

Supplemental discussion of *Cornus florida* findings

Looking comprehensively at our *C. florida* results, we see potential signals of complex dynamics linking numerous response and predictor variables, and spanning all analysis intervals. In the first summer (2018), *C. florida* seedlings exhibited greater foliar damage in more open locations and in areas with greater abundance of adult *C. florida* trees. However, in the same interval, *C. florida* seedlings grew faster in areas with greater canopy openness and higher conspecific abundance. Thus, two conditions that were positively associated with height growth (open canopy, proximate *C. florida* trees) were also associated with greater foliar damage. It is possible that positive plant-soil feedback helped with the early establishment of transplanted seedlings near adult conspecifics (Kardol et al. 2013), but that seedlings in these same locations experienced increased exposure to species-specific foliar pests and/or pathogens. Some of these agents may also benefit from a reduction in canopy cover, or perhaps inflict greater damage on faster growing seedlings (Züst and Agrawal 2017; Martini et al. 2021). No negative effects on growth or survival manifested that first summer, but height measurements the following year (2019) revealed that seedling growth was lower in areas with higher living conspecific abundance. As such, the foliar damage documented during the first summer may have led to reduced growth from 2018 to 2019. Furthermore, while nothing was predictive of *C. florida* survival from 2018

to 2019, living conspecific abundance was the sole significant predictor of survival from 2019 to 2020, and the association was negative. So, putting it all together, and aligning with previous studies that have detected conspecific inhibition in *C. florida* (Johnson et al. 2012; Bennett et al. 2017), one potential explanation is that young *C. florida* seedlings near conspecific adults are prone to foliar damage, which leads to reduced growth over the following year, and then to reduced survival rates the year after that. It is also possible that the stressful drought conditions of late summer 2019 acted in concert with lagging conspecific effects, and that the increased mortality by 2020 would not have occurred without the drought (Castagneyrol et al. 2018; Szczepaniec and Finke 2019;).

Digging deeper into the *C. florida* results, we can once again attempt to make sense of complex multi-year patterns, this time focusing on the ways in which drought may modify biotic neighborhood effects. Seedling height growth was negatively affected by living conspecific and heterospecific abundance prior to the drought, but both predictors became non-significant during the drought-impacted interval. This could be because the drought stress overwhelmed other inhibitive effects or – similarly – it could be that the lower growth rates simply made it more difficult to detect local neighborhood effects. We see a different pattern, however, for *C. florida* survival, with living conspecifics having no effect pre-drought, but then a negative effect on survival during the drought. This potential drought-mediated strengthening of conspecific inhibition is surprising given that several other studies have found drought-associated weakening (e.g., Newbery and Stoll 2013; Bachelot et al. 2015; Uriarte et al. 2018; Xi et al. 2022). However, it may be that the same negative conspecific mechanism (e.g., a species-specific foliar pathogen) manifests as slower growth in a non-drought year, but transitions into reduced survival in a more stressful, higher-mortality situation. Accordingly, it is possible that conspecific inhibition plays a temporally variable but important role in *C. florida* regeneration dynamics, perhaps helping to prevent the formation of a mono-dominant subcanopy layer.

Literature cited above that is not cited in main text

Bennett JA, Maherali H, Reinhart KO, et al (2017) Plant-soil feedbacks and mycorrhizal type influence temperate forest population dynamics. Science 355:181–184. https://doi.org/10.1126/science.aai8212

Castagneyrol B, Jactel H, Moreira X (2018) Anti-herbivore defences and insect herbivory: Interactive effects of drought and tree neighbours. Journal of Ecology 106:2043–2057. https://doi.org/10.1111/1365-2745.12956

Szczepaniec A, Finke D (2019) Plant-Vector-Pathogen Interactions in the Context of Drought Stress. Frontiers in Ecology and Evolution 7. https://doi.org/10.3389/fevo.2019.00262

Züst T, Agrawal AA (2017) Trade-Offs Between Plant Growth and Defense Against Insect Herbivory: An Emerging Mechanistic Synthesis. Annual Review of Plant Biology 68:513–534. https://doi.org/10.1146/annurev-arplant-042916-040856
